# Diagnosis and Treatment of Nodular Fasciitis of Ear Region in Children: A Case Report and Review of Literature

**DOI:** 10.3390/healthcare10101962

**Published:** 2022-10-07

**Authors:** Antonio Della Volpe, Paola Festa, Alfonso Maria Varricchio, Carmela Russo, Eugenio Maria Covelli, Delfina Bifano, Piera Piroli, Antonietta De Lucia, Arianna Di Stadio, Franco Ionna

**Affiliations:** 1 Otolaryngology Department, Cochlear Implant Regional Referral Center, Santobono-Pausilipon Children’s Hospital, 80129 Naples, Italy; 2 Neuroradiology Department, Santobono-Pausilipon Children’s Hospital, 80129 Naples, Italy; 3 Department of Pathology Santobono-Pausilipon Children’s Hospital, 80129 Naples, Italy; 4 Department GF Ingrassia, University of Catania, 95131 Catania, Italy; 5 Maxillofacial and ENT Surgery Unit, Fondazione Pascale Hospital IRCCS, 80131 Naples, Italy

**Keywords:** nodular fasciitis, external ear, parotidectomy, myocutaneous flap, surgery, child

## Abstract

Nodular fasciitis (NF) is a benign fibroblastic and myofibroblastic proliferation of subcutaneous tissues. Rarely, it has been identified in the ear and more rarely in children. We describe a case in a four-year-old girl and the surgical management of it. The patient was referred to the otolaryngology unit of a tertiary referral center because she was affected by a painless and growing lesion in the left external auditory canal (EAC). The girl was treated by large-spectrum antibiotic therapy for one week without success. For this reason, we requested ultrasonography (US) of the left hemiface, maxillofacial and temporal bone computed tomography (CT) and magnetic resonance imaging (MRI) of the head with and without contrast. The imaging identified an irregular ovoid hypoechoic nodule with distinct margins indissociable from the cartilaginous planes and extending into the parotid loggia with local infiltration of the fascia. The lesion was surgically removed through preauricular access due its extension into the parotid area. The mass was excised in toto and sent to the pathologist for immunohistochemistry. The histopathologist based on the finding diagnosed a nodular fasciitis. In case of suspicion of malignancy, early investigations should be done to evaluate the lesion, then a traditional parotidectomy can be safely and successfully performed even in a very young child. The open technique allows the removal of NF with full control of the surgical area and facial nerve. In this article, we presented the management of a case in a 4-year-old female affected by NF of the external auditory canal (EAC), and we described clinical and surgical management of the case. We also reviewed literature of nodular fasciitis cases of ears in children.

## 1. Introduction

Nodular fasciitis (NF) is a benign fibroblastic and myofibroblastic proliferation of subcutaneous tissues firstly described by Konwaler et al. in 1955 [1]. NF was classified as a soft tissue tumor by the World Health Organization (WHO) in 2013 [2]. Its prevalence in children is approximately 10% [3]. Rarely NF localized in the ear region was reported in literature [4,5] and more rarely described in pediatric age [6,7]. Masses in the external ear can be due to rhabdomyosarcoma [8], angioleiomyoma [9], fibroepithelial polyp [10], squamous cell carcinoma [11], otitis malignant and suppurate tissue in case of persistence of an external body in the EAC [12]. The diagnostic management includes ultrasonography (US), computed tomography (CT) and magnetic resonance imaging (MRI) [7]. However, only histopathology can confirm the diagnosis [4]. After a short period of observation, the lesion must be surgically removed in toto [7]. Some authors proposed injection of intralesional steroids or partial removal of the lesion [6]. This case report aimed at presenting the clinical and surgical management of a case of NF in a 4-year-old female, who presented the lesion in the external auditory canal (EAC); moreover, we reviewed the literature to identify similar cases previously described.

## 2. Materials and Methods

### Search Strategy for Systematic Review of the Literature

To identify relevant studies of FN, we systematically searched MEDLINE/PubMed, EMBASE and Cochrane Central Register of Controlled Trials. The search strategy was carried out until 19 September 2022. Keywords were nodular fasciitis, ear and child. These keywords were used differently in the screened platform to maximize the identification of relevant articles. For PubMed we used: (‘nodular fasciitis’ (MesH Terms) AND ear (MesH Terms)), a filter by age “child:birth-18 years” was used. For EMBASE: ‘fascitiitis nodular ear children’ OR (‘fascitiitis’/exp OR fasciitis) AND (‘nodular’/exp OR nodular) AND (‘ear’/exp OR ear) AND (‘children’/exp OR children) was used. For Cochrane only, ‘nodular fasciitis’ was used for the search strategy.

Inclusion criteria were papers in English, case reports, longitudinal studies and clinical trials and children. No other restriction was applied.

Two researchers (P.F. and A.D.S.) reviewed the search results and screened titles of the search lists from the electronic sources. The duplicates were removed, and the abstracts were read in full to remove the studies outside the scope of the review. Additionally, a hand-search was performed to identify additional studies of interest.

Therefore, the full text of all potentially eligible studies was read by the two researchers, discrepancy was discussed with a third investigator (A.D.V.) to provide resolution and agreement between the two. The papers that did not fulfill inclusion criteria were excluded.

## 3. Case Presentation

**Figure 1:**
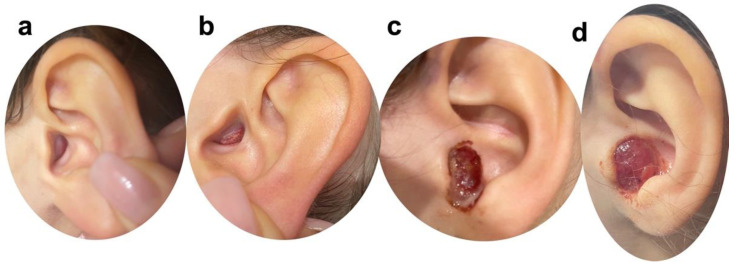
(**a**,**b**) Left ear. Clinical appearance of the mass at the first follow-up; (**c**,**d**) Clinical aspect of the mass after 1 week of unsuccessfully anti-biotic treatment.

A four-year-old female patient was referred to the Otolaryngology Unit of Santobono-Pausilipon Paediatric Hospital (Naples, Italy) because her parents noted a mass in her left EAC. On observation, a 5 mm skin-colored and painless lesion was identified (Figure 1a,b). No recent history of facial trauma or surgery was mentioned. As a first approach, in thinking about an insect bite, we prescribed oral treatment with amoxicillin/clavulanic acid (5 mL every 12 h), betamethasone sodium phosphate (0.5 mg daily) and local tobramycin (2 drops 3 times a day into the ear) for 7 days. At the control, one week after treatment, the lesion had a rapid growth and was occupying the entire auricular concha. The lesion, now 15 mm (1 cm), was purple-red colored, ulcerated and prone to bleeding (Figure 1c,d).

Routinary blood tests were normal. Because of the clinical finding and suspecting a malignity, we requested ultrasonography (US) of left hemiface, maxillofacial and temporal bone computed tomography (CT) and magnetic resonance imaging (MRI) of the head with and without contrast. We requested both imaging to evaluate bone infiltration (CT) and the relationship of the mass with the surrounding structures as vessels and nerves (MRI). US examination of the left auricle evidenced in the subcutaneous layer an ovoid formation of 12 mm of diameter with regular margins and inhomogeneous echostructure. This formation insinuates deeply along the external auditory canal for about 30 mm, and it shows contiguity with the parotid without infiltrated signs.

**Figure 2:**
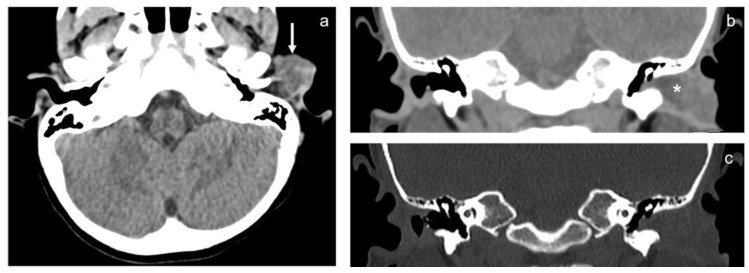
(**a**) CT axial view. White arrow shows the mass that extends outside the left EAC. (**b**) and (**c**) CT coronal view. * shows the mass.

**Figure 3:**
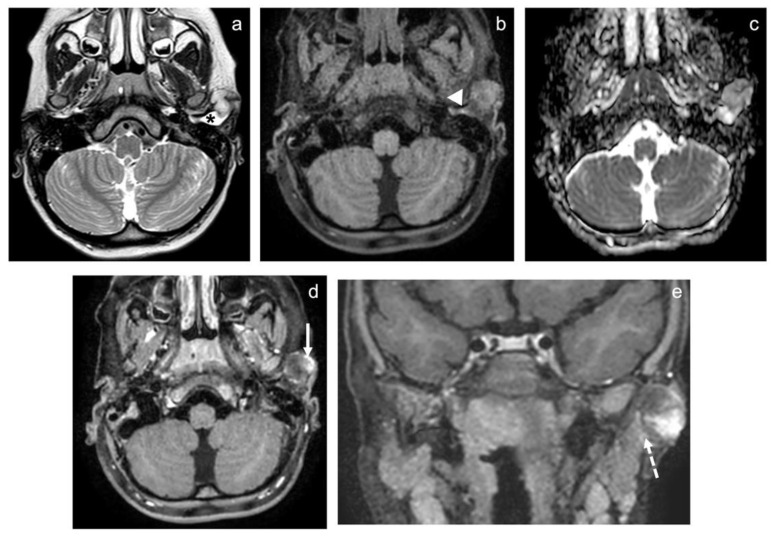
(**a**–**d**) Axial MRI FLAIR and traditional sequences. * and the white arrows show the mass and its relationship with surrounding tissue. (**e**) Coronal view shows (white interrupted arrows) the relationship of the mass with the parotid fascia and the gland.

CT and MRI showed a 17 × 21 × 24 mm lobulated and well-defined solid lesion, which involved the cartilaginous planes of the left tragus and the lateral portion of the external acoustic canal (EAC) (Figure 2 and Figure 3). It extended into the parotid space where it infiltrated the cutaneous and subcutaneous layers, compressing the parotid gland without infiltrating it. On MRI, the lesion is hyperintense on T2-weighted and isointense on T1-weighted images, compared with the adjacent muscles. Diffusion weighted imaging with ADC (apparent diffusion coefficient) maps revealed high-to-moderate diffusivity, suggesting the low cellularity of the lesion. After contrast administration, it showed irregular and peripheral enhancement. Fluid entrapment within the EAC was also associated, and there was no evidence of bone erosion.

**Figure 4:**
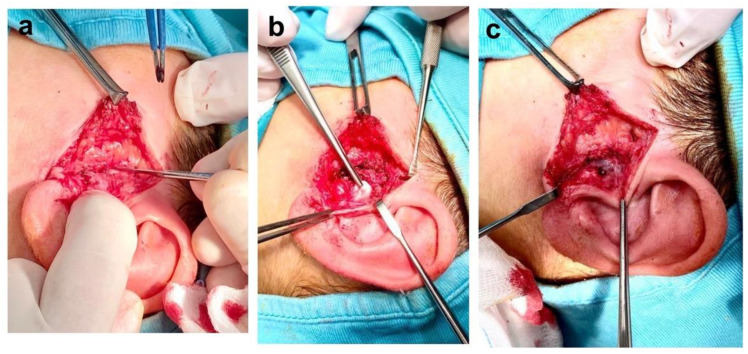
(**a**) Parotidectomy approach; (**b**) Identification of the mass; (**c**) Surgical area after removal of nodular fasciitis.

Surgical treatment was performed under general anesthesia and local infiltration of xylocaine+ adrenaline by left preauricular incision. A subcutaneous muscular flap was created and elevated exposing the parotid loggia, where the mass was clearly identified (Figure 4a–c). The lesion was encapsulated and not infiltrating the surrounding tissue. The neoformation was isolated from the cartilage of the tragus and the parotid gland by blunt dissection, then entirely removed with its capsule (Figure 1E). During the surgery, the integrity of the facial nerve was monitored and controlled by visual control and nerve stimulation by NIM (Medtronic, Jacksonville, FL, USA). The mass was sent to the pathologist for histologic diagnosis with immunohistochemistry (IHC) and fluorescence in situ (FISH). The patient was treated by intravenous ceftriaxone (0.80 mg/kg) and betamethasone sodium phosphate (0.5 mg/day) for 7 days; then topical therapy with tobramycin and dexamethasone (2 drops/3 times day) was prescribed for 12 days. No facial deficit was identified immediately after surgery and in the following controls.

**Figure 5:**
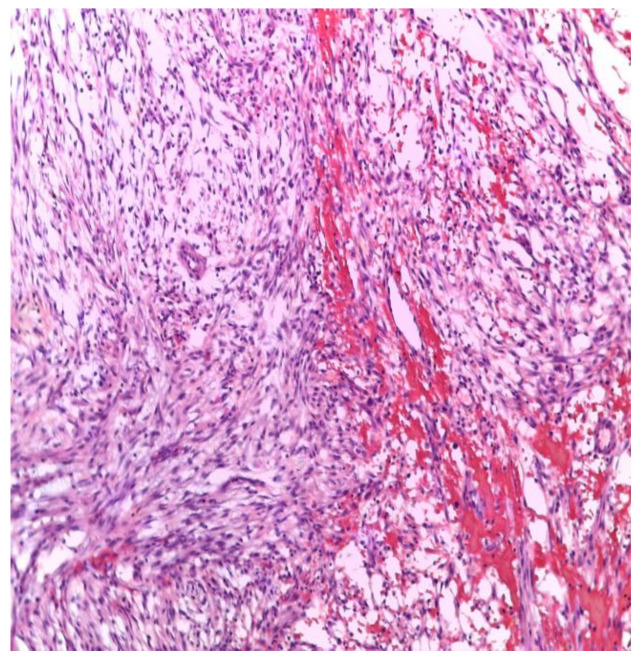
Histologic finding that shows characteristic aspects of nodular fasciitis: sheets and bundles of spindle cells with cystic myxoid changes and extravasated erythrocytes (H&E × 100).

The histological examination of the tissue hematoxylin and eosin (H&E) stained showed positivity for smooth muscle actin (SMA), vimentin, calponin, actin and negativity for caldesmone, S100, EMA, ALK, panCK, NSE, desmin, CD34. Fluorescence in situ hybridization (FISH) showed that 30% of the 100 cells exhibited ubiquitin-specific peptidase 6 (USP6) gene rearrangement. Based on these findings, the diagnosis of NF was established (Figure 5).

Three months after surgery, an MRI with and without contrast was performed on the patient that confirmed the complete resection of the lesion without any recurrence. The last follow-up (12 months after resection) showed no recurrence.

## 4. Discussion

Overall, our case showed that the traditional parotid approach can also be safely performed in children without increased surgical risk, and it can be very useful to correctly remove NF of the ear.

We identified only nine articles [4,6,7,13,14,15,16,17,18] from our research and inclusion criteria that can be discussed (Table 1).

The biggest study was the retrospective review of 50 cases of auricular NF done by Thompson et al. [4]; at presentation the mean age was 27 years but there was no info about the age at the onset of the disease. The remaining eight articles were two retrospective studies and six case reports, which described a total of thirteen pediatric patients (7 males and 4 females); two cases did not report sex [13,16]) from 17 months until 18 years old of age [6,7,8,13,14,15,16].

Two case reports described a postauricular NF in an 18-year-old male [14] and an NF in the temporal region in a 14-year-old girl [15].

NF in the external auditory canal was similarly identified by Thompson et al. [4] in six of the 50 cases included in the case series. NF of the ear is rare in such young children, anyway we identified in the literature other 11 children with NF in the same [6,7,13,16,17,18].

Presence or absence of pain is not distinctive of NF, in fact it can [6] or cannot be referred by the patients [17,18]. Abdel-Aziz et al. [6] described six Egyptian children (four males and two females, age range of 5–13 years) with NF of the EAC, which, in all cases, was accomplished with unilateral pain in the affected ear. The absence of pain was reported five pediatric cases previously described [7,13,16,17,18]. In our case, the lesion was initially painless and became painful after one week.

Bleeding, as well as pain, can be observed with different prevalence depending on the study and the age of patients included. Bleeding, as in our case, was reported in five children with NF of the external auditory canal [6,7,16].

In general, our clinical diagnostic and histological findings overlapped previous studies [6,7,9].

In literature there is a lack of description of the surgical approaches in children, and, to date, the most interesting paper was presented by Liu and Li [13]. The authors under general anesthesia removed in a 17-month-old child the mass “en bloc”, including the entire cartilage of concha given of tumor infiltration. The main risk of NF is recurrence due to incomplete resection or auricular trauma [7,13]. Incomplete resection could happen due to the anatomical position of the lesion that does not allow its complete access [7,13].

Because of the location and tumor infiltration, the recurrence because incomplete resection has been report in 33.3% of cases [6].

To maximize the results and improve the long-term free-of-disease period, different surgical approaches have been proposed based on the location and position of the lesion [7,8,13,14,15].

We used a typical “parotid gland approach” with myocutaneous flap that allowed a clear identification of the mass, its relationship with the surrounding structure, monitoring of the facial nerve by visual check and NIM stimulation and easy management of eventual emergency during surgery (i.e., vessel bleeding).

## 5. Conclusions

NF should be considered as a differential diagnosis when a mass originating from the EAC is identified. The use of a traditional surgical approach, as the one for parotidectomy, can also be safe in young children. In this population, this method allows not only a fully open view of the mass and its surroundings but also the monitoring of noble structure (VII c.n) upon which a lesion could be extremely disfiguring and disabling. Finally, this access allowed removal of the entire mass, and the patient was disease free at short and middle-term follow-ups.

## Figures and Tables

**Table 1:** Characteristics of nine studies included in the literature review.

References, Nation, Year	Type of Study	Sample Size	Age (Mean, Range; Age), Gender	Location (Number of Patients)	Pain (Number of Patients)	Bleeding (Number of Patients)	History of Trauma (Number of Patients)	Diagnostic Imaging	Surgery	Follow Up (Years)/ Recurrence (Number of Patients)
**Thompson et al., United States of America, 2001** [4]	Original article, Retrospective	50	27.4 (1–76) years 28 M/22 F	Ear, not otherwise specified (5)	5	5	5	Not reported	Not described	13.4 years/Yes (4)
				External auditory canal (6)						
				Pinna (2)						
				Preauricular (18)						
				Posterior auricular (19)						
**Huang et al., China, 2007** [14]	Case report	1	18 years 1 M	Posterior auricular	Not reported	No	No	Computed Tomographic	Y-shape incision along the earlobe	3.0 years/No
**Abdel-Aziz et al., Egypt, 2008** [6]	Original article, Retrospective	6	7.4 (5–13), years 4 M/2 F	External auditory canal	6	3	None	Computed Tomography	Through the external auditory canal	1.0 years/Yes (2)
**Jovanovic et al., Serbia, 2012** [15]	Case report	1	14 years; 1 F	Temporal region	No	No	No	Computed Tomographic	Not described	2.0 years/No
**Barbot et al., United States of America, 2019** [17]	Case report	1	12 years; 1 M	External auditory canal	Not reported	Not reported	Yes	Computed Tomographic, Magnetic Resonance Imaging	Firstly, incisional biopsy; surgical local excision with exposure of tragal and conchal bowl cartilage	0.5 years/No
**Hasley et al.,** **United States of America, 2020** [16]	Case report	1	19 months; sex not reported	External auditory canal	No	Yes	Yes	Not reported	Radical surgical resection of the entire conchal bowl	Not reported/No
**Liu and Li, China, 2021** [13]	Case report	1	17 months; sex not reported	External auditory canal	No	No	Not reported	Regular and Enhanced Magnetic Resonance Imaging	Auricular mass resection and free flap implantation	1 year/No
**Cabanoglu et al., Turkey, 2021** [18]	Case report	1	8 years; 1 M	External auditory canal	Not reported	Not reported	Yes	Enhanced Computed Tomographic	Firstly, excisional biopsy; postauricular approach after one month	0.6 years/No
**Wang et al., China, 2022** [7]	Original article, Retrospective	3	24 (17–36) months 1 M/2 F	Preauricular (1)	No	1	None	Plain Computed Tomography (3); Enhanced Magnetic Resonance Imaging (2); Enhanced Computed Tomography (2); Regular Magnetic Resonance Imaging (2) Ultrasonography (3)	Longitudinal incision along the preauricular bulge(1); fusiform incision on the posterior aspect of the pinna (1); surgical incision along the edge of the mass	1.3 years/None
				Pinna (1)						
				External auditory canal (1)						

## Data Availability

Original data are available under request to the corresponding author.
